# Effects of cavity orientation on nesting success inferred from long-term monitoring of the endangered red-cockaded woodpecker

**DOI:** 10.1038/s41598-022-15201-x

**Published:** 2022-07-08

**Authors:** Lukas Landler, James Skelton, Michelle A. Jusino, Andy Van Lanen, Jeffrey R. Walters

**Affiliations:** 1 Department of Biological Sciences, Virginia Tech, 926 West Campus Drive, Blacksburg, VA USA; 2 Institute of Zoology, University of Natural Resources and Life Sciences (BOKU), Vienna, Austria; 3 Department of Biology, College of William and Mary, Williamsburg, VA USA; 4 USDA, Forest Service, Northern Research Station, Center for Forest Mycology Research, Madison, WI USA; 5 Florida Fish and Wildlife Conservation Commission, Three Lakes WMA, Kenansville, FL USA; 6 Present Address: Sandhills Ecological Institute, 515 Midland Road #E, Southern Pines, NC USA

**Keywords:** Ecology, Evolution, Ecology, Environmental sciences

## Abstract

Animals that create structures often display non-random patterns in the direction of their constructions. This tendency of oriented construction is widely presumed to be an adaptive trait of the constructor’s extended phenotype, but there is little empirical support for this hypothesis. Particularly, for cavity nesting-birds there is a lack of studies examining this issue. In this study of a primary cavity excavator, the endangered red-cockaded woodpecker (*Dryobates borealis*), we show that cavity entrances exhibited a strong westward bias in all 11 of the populations examined throughout the geographic range of the species in the southeastern United States. This species requires cavities in living pine trees for roosting and nesting that often take many years to complete, resulting in many incomplete excavations on the landscape. We used population monitoring data to show that orientation was stronger among completed cavities than incomplete cavities. There was a significant correlation between latitude and average cavity direction among populations, turning northward with increasing latitude, suggesting adaptation to local conditions. Long-term monitoring data showed that cavity orientation and breeding group size are correlated with egg hatching rates, fledging rates, and the total number of fledglings produced per nest. Our results provide empirical evidence from extensive long-term data that directional orientation in animal constructions is an important feature of the extended animal phenotype and have immediate implications for animal ecology and the conservation of endangered species.

## Introduction

Many animals such as termites, spiders, fish, and birds build structures that serve important functions^1–4^. Such structures are an extension of the constructors’ phenotypes^5^. Features of animal structures could promote the builder’s fitness by reducing the vulnerability of the builder to the environment or predators, or by increasing the survival of the builder’s offspring. Furthermore, animal constructions alter the physical environment, which in turn imposes selection on the constructors, leading to important feedbacks that may extend to other members of surrounding ecological communities^6,7^.

Members of the avian family Picidae (woodpeckers and allies) characteristically excavate cavities in the wood of living and dead trees. These excavations provide shelter from external environmental conditions and protection from predators for eggs, nestlings, and roosting adults. Cavity construction is a considerable expense of time and energy^8,9^. Some species excavate a new nesting cavity each year, whereas others may use completed cavities for multiple breeding seasons^9^. Because cavity excavation is costly and excavated cavities are a valuable resource, behaviors that result in differences in cavity quality are likely to be under significant natural selection. Orientation of woodpecker cavities typically is non-random, suggesting that orientation may be a component of cavity quality^10,11^. Furthermore, the mean cavity direction of excavator species and populations varies according to latitude, at least in the northern hemisphere, suggesting that cavity orientation is adapted to local conditions^10^. Hypotheses to explain cavity orientation have focused primarily on factors that affect nest microclimate such as exposure to temperature, moisture and wind^12,13^. Wiebe^12^ found no relationship between reproductive success and cavity orientation, whereas Hooge et al.^14^ indicated that successful reproduction was related to cavity orientation. However, in-depth analysis of the influence of cavity directions on measures of fitness are still lacking for woodpeckers. There are numerous publications investigating nest directionality in other birds, however, while the effect of direction on microclimate seems clear (for a recent example see Schaaf and de la Pena^15^), its effect on fitness is less obvious and might not (always) be related to temperature^16–22^. Furthermore, in contrast to most other studies investigating nest orientation fitness effects, where secondary cavity-nesters choose already existing cavities [e.g.,^17^], woodpeckers themselves determine the directionality of their cavity.

Using an analysis of literature data and long-term monitoring data, we investigated cavity orientation and its relationship to reproductive success in the federally endangered (USA), cooperatively breeding red-cockaded woodpecker (*Dryobates borealis*; RCW). RCWs are particularly interesting for studies of cavity orientation for several reasons. Cavities are uniquely important to RCWs and cavity excavation, because it occurs exclusively through the sapwood and into the heartwood of living pine trees (*Pinus *spp.) and is an especially costly process that often takes years to complete^23,24^, rather than days to weeks as in most woodpeckers. During the prolonged excavation process, in which all adult group members participate, RCWs face the decision to continue, delay, or cease excavation of each cavity in progress. Once completed, cavities may be used for an equally long period^25^. Each group of RCWs maintains a year-round territory containing a “cluster” of trees with complete and incomplete excavations. RCWs use their cavities for nightly roosting; each bird in a group has its own complete roost cavity. Typically, the breeding male’s roost cavity is used as the nest tree for that year. Explanations for cavity orientation in this species include: taking advantage of fungal decay that may reduce excavation effort^26,27^, protection against rat snakes resulting from enhanced resin flow due to solar radiation^27–29^, and regulating nest microclimate factors such as moisture and temperature^30^.

We tested three predictions from the hypothesis that cavity orientation is a locally adapted feature of the woodpecker extended phenotype. (1) We predicted that cavity direction of RCW populations would have non-random unimodal distributions and the distributions of cavity directions would become less randomly distributed at each successive step in cavity construction as the birds abandoned poorly aligned cavities. We tested this prediction using extensive cavity orientation data from three geographically separated populations, comparing the strength of orientation in completed, incomplete, and nest excavations. (2) We predicted that cavity orientation would change with local climate and tested our prediction using a combination of published and unpublished data from 11 study populations and correlated the mean cavity direction of each population with latitude and local climate variables. (3) We predicted that cavities with the preferred cavity direction would also have the highest reproductive success. To test this prediction, we analysed 13 years of nesting data from one population using generalized linear and mixed effects models to assess the effects of cavity orientation and other factors on reproductive success at the egg, hatchling and fledgling stages.

## Methods

### Data collection

We analyzed comprehensive excavation entrance alignment data for three populations of RCWs: Marine Corps Base Camp ‘Lejeune’ in eastern North Carolina, USA, (lat: 34.60° long: − 77.37°, year: 2012), the ‘Sandhills’ in south-central North Carolina, USA (lat: 35.22° long: − 79.41°, year 2012) and ‘Three Lakes’ in southern Florida, USA (lat: 27.95°, long: − 81.14°; year: 2014). Although each of these sites has some human-made cavities constructed for RCWs as a conservation measure^31^ in addition to RCW-made excavations, only RCW-made excavations were included in this analysis. Excavation entrance alignments were measured using a handheld compass and the stage of each excavation was noted—completed excavations are termed ‘completed cavities’, excavation initiations and incomplete cavities are termed ‘cavity starts’. Complete cavities may be used by RCWs as roosts, and some are also used as nests. Cavities containing a RCW nest in the study year were recorded as ‘nest’. Mean vectors for each distribution of cavity directions were calculated using vector addition. In this calculation, each cavity orientation represents a vector with the length (r) of 1; the vector sum is then normalized for the number of data points. Therefore, the vector length of the mean vector can be between 0 and 1, where 1 constitutes a perfectly clustered orientation (all vectors are pointing in the exact same direction).

To explain geographic variation in excavation orientations among discrete woodpecker populations, we examined population level patterns in RCW excavation orientation taken from published and unpublished sources. We re-analyzed all published data concerning RCW cavity alignment that we could locate through an extensive search using citations in previous papers [e.g.^10,32^] and literature searches on Google scholar (Table 1). Data were taken from tables or figures. Mean vectors were calculated for every study with individual data points reported. For studies where data were collected from more than one site, we approximated a spatial mean by calculating the means of given latitudes and longitudes. Published data were combined with the data sets from our three study sites and an additional previously unpublished data set from Osceola County, Florida (coordinates: lat: 28.06°, long: − 80.97°).

**Table 1:** Collection of literature data and data presented in the current study sorted by latitude.

Study	State	Latitude	Longitude	Orientation	r statistic
Three Lakes (current study)	FL	27.95	− 81.14	255	0.47
Osceola (current study)	FL	28.06	− 80.97	242	0.45
Baker^27^	FL	30.66	− 84.21	243	0.39
Lay^33^	TX	31.45	− 95.43	265	0.29
Jones and Ott^34^	GA	32.89	− 84.18	250	0.54
Hopkins and Lynn Jr^35^	SC	33.8	− 80.72	271	0.34
Dennis^28^	SC	33.82	− 78.68	279	0.55
Wood^36^	OK	34.5	− 94.64	290	0.45
Lejeune (current study)	NC	34.6	− 77.37	265	0.42
Sandhills (current study)	NC	35.22	− 79.41	269	0.27
Kalisz and Boettcher^37^	KY	36.8	− 84.3	285	0.28

The mean orientation, mean vector length (r), latitude, and longitude in decimal degrees are given for each data set. For all tests of non-random orientation in all populations, p ≤ 0.001.

Reproductive data were collected from the Lejeune population during 1999–2012. Methods for monitoring reproduction are described in detail in Walters et al.^38^. Briefly, all RCW territories on the base were visited in April of each year to determine if they were occupied by a RCW family group. Occupied territories were then visited every seven days to check for the presence of a nest using a Tree-top Peeper (Sandpiper Technologies, Manteca, CA, USA) or climbing with Swedish ladders. When nests were discovered, they were revisited every seven days until nestlings were old enough to be banded (i.e., age 6–10 days). Nestlings then were extracted from the cavity, fitted with unique combinations of coloured leg-bands and a United States Fish and Wildlife Service (USFWS) aluminium band, and placed back in the cavity. Groups were followed shortly after the projected fledging date to determine which of the banded young had fledged.

### Data analysis 1—do RCWs have a preferred cavity orientation?

To determine if each study population had a preferred cavity direction, Rayleigh tests were used to test for a unimodal distribution, as opposed to a random distribution of cavity directions^39^. We used a null modelling approach to test the prediction that cavity orientation becomes stronger at each step from cavity initiation (“cavity starts”) to nesting, as RCWs choose which cavities to complete, and in which to nest. We compared the observed directional biases of completed cavities to a null distribution generated from randomized subsets of the orientations of all excavation types—completed cavities and (incomplete) cavity starts. For each field site, we determined the degree of directional bias in completed cavities by calculating the mean resultant vector length (r) of all completed cavities (using function rho.circular, package “circular”, version 0.4-7 for R version 3.1.3^40^). We then created a null distribution of r by iteratively resampling with replacement the orientations of all excavation types 1000 times. The size of each resampling (n) was equal to the number of completed cavities observed at each site. Therefore, the null distribution for each field site represents the null expectation for r when completed cavities represent a random subset of all excavation types. Alternatively, if the observed r was found to be significantly greater than null expectations (one-tail test, α = 0.05), we concluded that completed cavities represented a significantly more clustered subset of all excavation types than expected by chance. We used similar methods to determine if RCWs had a directional bias in their selection of nesting cavities from all available completed cavities. Using the same approach, we also tested if cavity starts were significantly less clustered than the overall cavity distribution.

### Data analysis 2—is cavity direction adapted to local conditions?

We used linear regression to examine the relationship between mean cavity orientation (degrees) of each population as the response variable, and latitude (degrees), and the first two principal components of 19 climate variables for the location of each RCW population as predictor variables. We treated the circular response variable of mean orientation as a continuous linear variable because all observed values fell between 240° and 290°. Climate data for each RCW population were retrieved from http://biogeo.ucdavis.edu/data/climate/worldclim/1_4/grid/cur/bio_2-5m_bil.zip (access date: January 18 2015, function getData, raster package for R^41^). The climate data variables were; annual mean temperature, mean diurnal temperature range, isothermality (mean diurnal range/annual range), temperature seasonality (standard deviation), maximum temperature of warmest month, minimum temperature of warmest month, annual temperature range, mean temperature of wettest quarter, mean temperature of driest quarter, mean temperature of warmest quarter, mean temperature of coldest quarter, annual precipitation, precipitation of wettest month, precipitation of driest month, precipitation seasonality (coefficient of variation), precipitation of wettest quarter, precipitation of driest quarter, precipitation of warmest quarter, and precipitation of coldest quarter (details may be found here; https://test2.biogeo.ucdavis.edu/wc2/#/bioclim). We then used principal components ordination (function princomp^42^) to create composite climate variables. Climate variables were centered on zero and scaled to number of standard deviations prior to ordination. We also determined if variation in local climate variables explained variation in cavity orientation that was not explained by latitude alone. This was done by creating three distance matrices; a response matrix consisting of pairwise differences in mean direction among all sites, a predictor matrix consisting of the pairwise Euclidean distances in the scaled and centered climate variables among sites, and a third matrix consisting of the pairwise differences in latitude among all sites. We then used a permutations based Partial Mantel test (two-tail test, 1000 permutations; function mantel; ecodist package for R^43^ to look for a significant correlation between the response matrix and the climate matrix, after removing the variation explained by the latitudinal matrix. We did not include altitude in this analysis because most of the variation in altitude in our data occurs within rather than between populations. Most populations are within the Coastal Plain where there is relatively little variation in elevation either within or between populations. The three exceptions (OK, KY, NC—Sandhills in Table 1) are inland populations of higher average elevation with hilly to mountainous terrain.

### Data analysis 3—does nest cavity orientation affect reproductive success?

We used generalized linear and mixed effects models of the Lejeune long-term nesting data to assess the effects of nest cavity direction on reproductive success. When family groups attempted multiple broods in a year, only the first attempt was considered. We used a Poisson model to assess effects on total nest fecundity measured as number of fledglings produced. Binomial models were used to assess effects on hatch rate (proportion of eggs that produced hatchlings) and fledge rate (proportion of hatchlings that recruited to fledglings). Effects of direction were modeled as paired sine and cosine terms, in order to account for the properties of circular data (i.e., 1° is closer to 360, than 270° on a circle). Initial models included sine and cosine terms for 1–4 cycles following Cox^44^. We also considered the number of adults in the family group as main fixed effects and interactions with the trigonometric terms. We considered cluster ID as a random factor to account for repeated measures within each cluster^45^. The best models were chosen and validated using a top-down approach following Zuur et al.^46^ section 5.8.2.4 using the glmer function in the lme4 package v1.1-20^47^.

### Ethical approval

Data collection was approved by IACUC 13-177-BiOL from Virginia Tech, the Endangered Species permit TE-070846 from the U.S. Fish and Wildlife Service, the Federal Banding permit 21544 from the U.S. Geological Survey and followed all relevant guidelines and regulations. The current study is based on observations, no experimental treatments were applied.

## Results

### Data analysis 1—do RCWs have a preferred cavity orientation?

**Figure 1:**
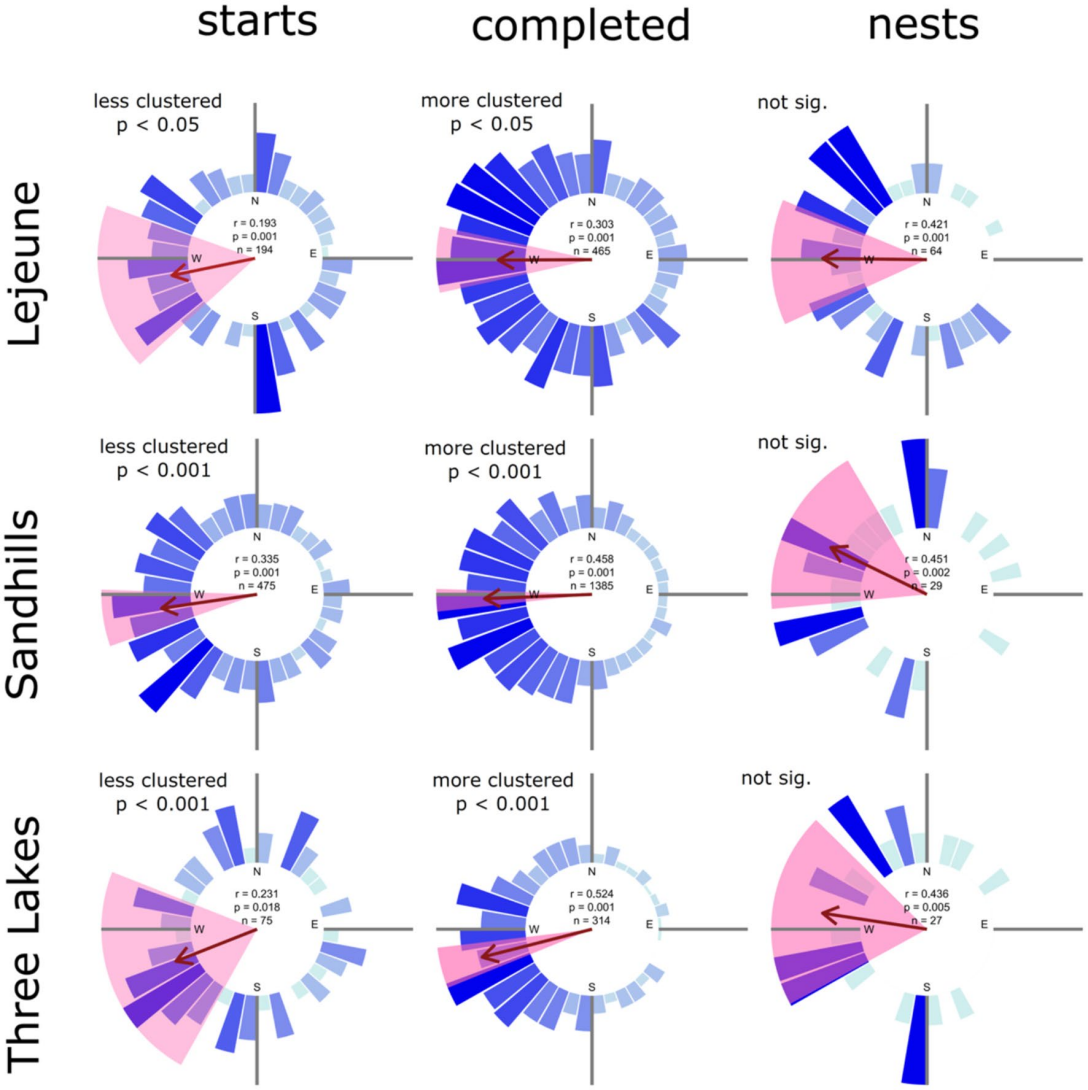
Three populations of Red-cockaded woodpeckers show significant preference for western cavity orientation at three excavation stages. Blue bars represent number of cavities within 10° increments. Red arrows show mean direction with length scaled to the r-statistic. Shaded wedges show boot-strapped 95% confidence intervals. Results of Rayleigh-tests of non-random orientation are provided in the centers of the compass roses. Null model comparisons showed that cavity starts are less clustered, and completed cavities are more clustered than the distributions of all cavities within each site. Nests were significantly oriented to the west at all sites, but not significantly more clustered than completed cavities at any site. P values for null model comparisons are given beside the compass roses.

RCWs prefer to excavate on the western facing sides of trees. Cavity orientations of RCWs in the four previously unpublished data sets and all literature accounts had highly significant biases in cavity direction, generally favoring west (Table 1). Moreover, RCW cavity orientation became increasingly directed during the excavation process. In each of the three study sites, the cavity distributions of completed cavities were significantly more clustered than null distributions drawn from all cavities (complete and incomplete), and incomplete cavities were significantly less clustered than null distributions (Fig. 1), indicating that cavities with the preferred orientation are more likely to be completed than cavities with the less preferred orientations. Although cavities that were used for nesting were also significantly oriented westwards in all three cases, they were not significantly more clustered than the complete cavities at any of the three sites (Fig. 1).

### Data analysis 2—is orientation direction adapted to local conditions?

**Figure 2:**
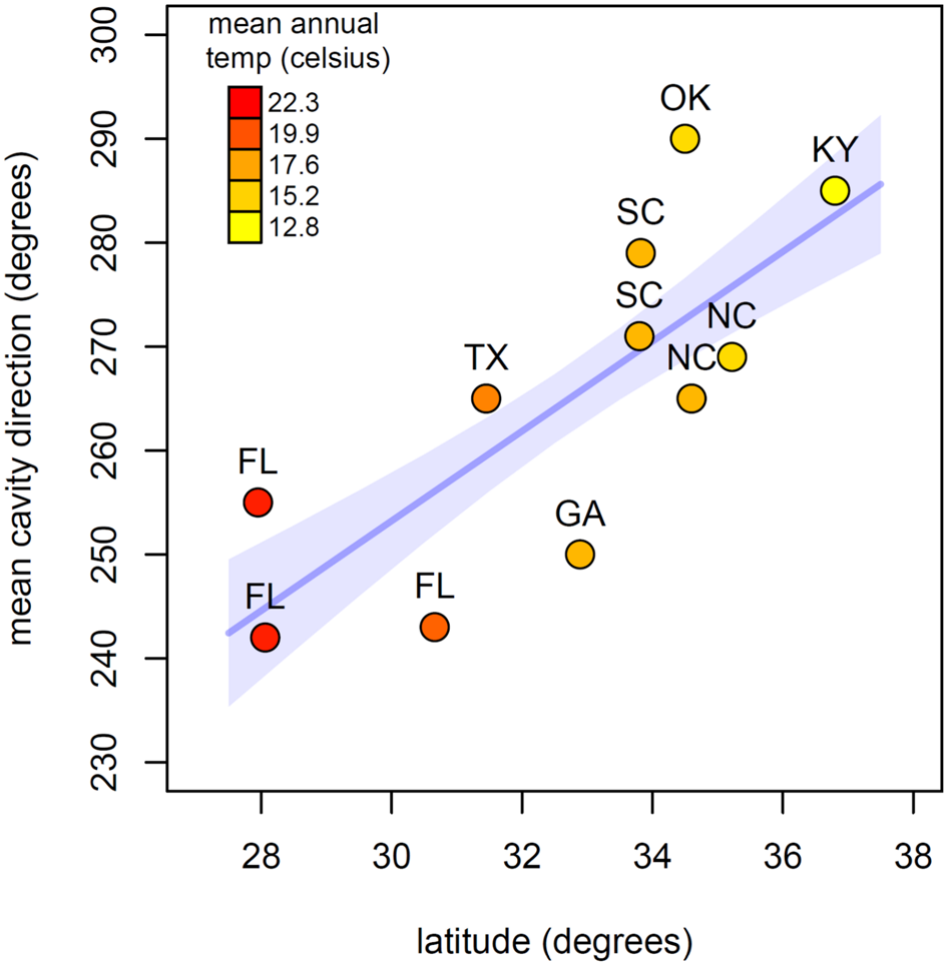
Cavity alignment significantly shifts toward north with increasing latitude (R^2^ = 0.588, t = 3.586, p = 0.006) and decreasing temperatures. Blue line and shading show model fit ± 1 SE. Data from studies reported in Table 1. Letters indicate the state in which the population is located.

Among the eleven RCW populations examined, mean cavity orientation was increasingly northward with increasing latitude (linear regression: effect of latitude, df = 9, t = 3.586, p < 0.006, R^2^ = 0.588; Fig. 2). The first principal component of local climate data (PC1) was also a strong predictor of mean cavity orientation (linear regression: effect of PC1, df = 9, t = 3.213, p < 0.011, R^2^ = 0.534). PC1 captured 88% of the total variance of the multivariate climate data set. Of all climate variables included, PC1 was most strongly correlated with minimum temperature of the coldest annual quarter (r = − 0.994), minimum temperature of the coldest month (r = − 0.992), temperature seasonality (r = 0.980), and annual mean temperature (r = − 0.979). Contrary to our expectations and previous work^10^, these correlations show that excavation orientation became more northward with colder temperatures and stronger seasonality. We could not separate the effect of local climate variables from purely latitudinal effects because latitude and PC1 were very strongly correlated (r = 0.961). Our multivariate analysis also could not distinguish latitudinal from climatic effects on mean cavity orientation. Once the variance due to differences in latitude was accounted for, Euclidean distances of climate variables did not explain a significant portion of the remaining variance (mantels r = − 0.064, p = 0.628).

### Data analysis 3—does nest cavity orientation affect reproductive success?

**Figure 3:**
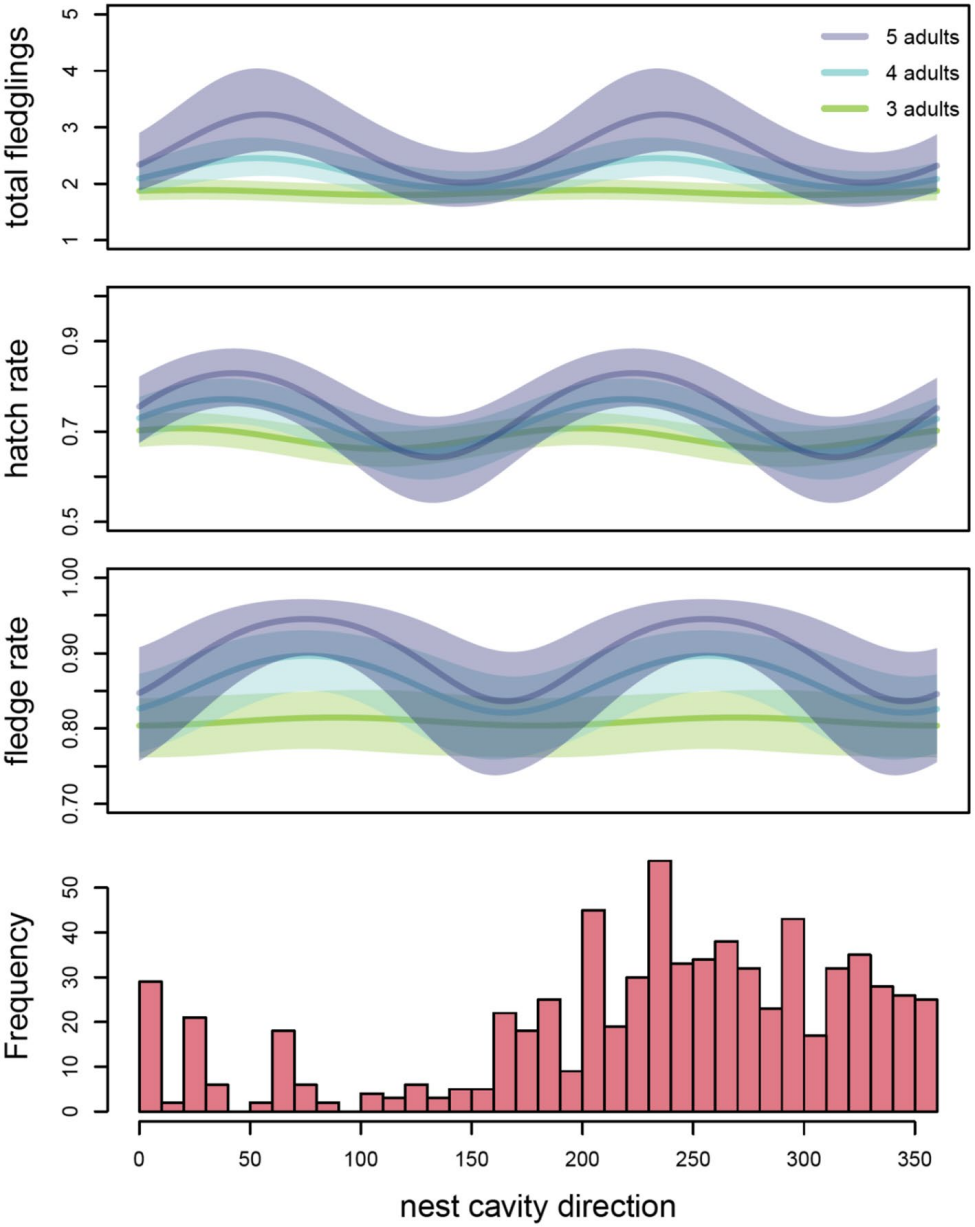
Nesting success of Red-cockaded Woodpeckers predicted by cavity direction (in degrees, x-axis) and the number of adult birds in the breeding group (by color). Top panel shows predicted total number of fledglings produced. Hatch rate is the predicted proportion of eggs that produced hatchlings. Fledge rate is the predicted proportion of hatchlings that will fledge. Lines represent GLMM fits for fixed effects, shading shows 95% confidence interval. GLMM details in Table 2. Bottom panel shows frequency histogram of nest cavities used (cavities used *n* times are counted *n* times)*.*

For all three analyses of reproductive success in the Camp Lejeune population—total fecundity, hatch rate, and fledge rate—cavity direction was a significant predictor of nesting success. Modeling results indicated that there were two directional optima for each breeding success measure and that the effects of nest direction were contingent on group size, with stronger effects of direction in larger family groups (Fig. 3). In each case, the final model included group size, 2-cycle sine and cosine terms, and the interaction terms (Table 2). All other trigonometric terms were dropped during model selection. The model for total fecundity was simplified to a generalized linear model because estimates of variance for cluster ID were approximately zero, resulting in singularity. Parameter estimates for fixed effects were the same for GLM and GLMM fecundity models.

**Table 2:** Generalized linear and mixed effects models showing main and interactive effects of breeding group size (number of adult birds) and nest cavity direction on breeding success.

Predictors	Total fledglings			Hatch rate			Fledge rate		
	Incidence rate ratios	CI	p value	Odds ratios	CI	p value	Odds ratios	CI	p value
(Intercept)	1.13	0.93–1.37	0.207	1.37	0.99–1.89	0.059	1.29	0.79–2.12	0.309
Adults	1.18	1.11–1.25	** < 0.001**	1.17	1.05–1.30	**0.004**	1.49	1.26–1.75	** < 0.001**
sin(2θ)	0.76	0.58–1.00	0.052	0.57	0.36–0.89	**0.015**	0.65	0.32–1.31	0.229
cos(2θ)	1.20	0.92–1.57	0.181	1.18	0.75–1.83	0.475	2.05	1.07–3.92	**0.031**
Adults: sin(2θ)	1.10	1.01–1.21	**0.033**	1.24	1.06–1.44	**0.007**	1.15	0.90–1.47	0.250
Adults: cos(2θ)	0.95	0.87–1.03	0.181	0.97	0.84–1.12	0.707	0.78	0.63–0.96	**0.022**
**Random effects**									
σ^2^				3.29			3.29		
τ_00_				0.06_Cluster_			0.17_Cluster_		
ICC				0.02_Cluster_			0.05_Cluster_		
Observations	702			702			702		
Cox and Snell's R^2^/Nagelkerke's R^2^	0.048/0.094			0.011/0.030			0.042/0.090		

Nest direction is represented by θ for the angular direction of the nest in radians. The total number of fledglings was modeled by GLM assuming a Poisson error distribution. Hatch rate and fledge rate were modeled by binomial GLMM.

Significant values are in bold.

## Discussion

This study is the most extensive test to date of the hypothesis that nest cavity orientation is an adaptive trait in the extended phenotype of cavity excavators. We found support for all three of our predictions that stemmed from this hypothesis. Cavity direction was strongly non-random in all of the populations we analysed and became more strongly oriented at successive stages of cavity construction. Cavity orientation was significantly correlated with climate and latitude, suggesting that local conditions determine the optimal cavity direction. Most importantly, cavity direction was significantly correlated with reproductive success. Together these results exemplify the importance of cavity direction to excavator reproductive success. However, the mechanism(s) behind cavity orientation and its relationship with reproductive success remain elusive.

All of the analysed RCW populations showed clear westerly cavity entrance orientations. The strength of orientation is consistent across study sites throughout the RCW range, though cavity entrance orientation changes significantly with latitude. Although all populations prefer a westward orientation, there was a clear shift with increasing latitude to a more northerly cavity alignment (Fig. 2). This latitudinal trend in cavity orientation is inconsistent with the hypothesis that the impact of solar radiation on cavity microclimate drives orientation^12,34^. The correlation with latitude is opposite to what has been observed in other temperate zone systems where more northerly populations had more south-facing orientations, presumably to compensate for the cooler climate^10^. Note also that the few elevational differences that exist in these data reinforce this conclusion as the populations in which RCWs occur at the highest altitudes (OK, KY in Fig. 2) are among the northernmost populations. The pattern is also inconsistent with the hypothesis that cavities are oriented to provide protection from rain or wind as the prevailing winds are from the west/southwest in the south-eastern US. Because climate variables and latitude are strongly correlated, we could not determine whether climate variables or other factors associated with latitude, such as changes in directional cues, drive the variation in mean cavity orientation observed across woodpecker populations.

Thus, our results suggest that benefits other than temperature impacts on microclimate in the cavity are the most important determinants of cavity orientation in RCWs. One possible proximate explanation is that the sun could influence excavation orientation as a directional cue. If the birds orient to the sun’s position at sunset, they would construct their cavities with a generally westward alignment. However, the position of the sun at sunset changes throughout the year in a manner that varies with latitude. RCWs excavate cavities primarily during the summer months after the nesting period. If the excavation period occurs later at more northern latitudes, as nesting does, one may expect cavity alignment to shift northward at more northern locations as the position of the sun at sunset shifts. However, the expected change due to the sunset azimuth per latitudinal degree based on the timing of nesting seasons at different latitudes would be much smaller in magnitude (about tenfold less) than the change in orientation actually observed. Therefore, this hypothesis does not sufficiently explain the latitudinal variation we observed.

The particular cavity orientation of RCWs may relate to peculiarities of their biology. RCWs create and maintain small wounds, termed resin wells, in the tree cambium around the cavity entrance. These holes exude a barrier of sticky resin around the cavity^48^ that prevents predatory snakes from reaching the cavity^49^. A western exposure to evening sun is thought to increase resin flow just before evening when the birds go to roost^32^. However, this hypothesis does not explain the latitudinal variation in orientation we observed. Another possibility is that certain fungal communities might be positively influenced by the western direction and facilitate the lengthy excavation process^50–52^. For instance, in red-naped sapsuckers (*Sphyrapicus nuchalis*) cavity orientation might be related to fungal activity^53^.

Long-term breeding data from Marine Corps Base Camp Lejeune revealed significant correlations between nest cavity direction and all three measures of reproductive success considered: total fledglings, hatch rate, and fledge rate. The interaction between RCW group size and cavity direction indicates that optimal cavity orientation is only beneficial to larger breeding groups. Larger groups contain more non-breeding adult helpers that have been previously shown to improve reproductive success^54^. Part of this benefit is because helpers buffer various aspects of reproduction from environmental factors that have adverse effects on groups without helpers^55,56^. It may be that these other factors mask the benefits of cavity orientation in smaller groups. For example, larger groups might benefit from western cavity orientations more than smaller groups because they have a sufficient labour force to maintain resin wells to protect nests from snakes.

Predictions from all three models indicated two directional optima 180 degrees apart (W and E), with one coinciding with the generally preferred western orientation. This suggests that RCW reproduction is equally successful when nests are excavated on the eastern or western facing sides of the tree, though the eastern side is rarely used. In migratory birds it has been shown that the offspring of parents with different directional preferences often exhibit intermediate orientations [e.g.^57,58^]. In the case of RCWs, such an intermediate directional preference (towards N or S) would result in decreased offspring survival. Therefore, selection may drive the population towards one of the two fitness maxima (E or W). This might be facilitated by another factor independent of offspring survival, which provides a small benefit for excavating towards West over East (e.g. fungal growth, wood hardness and a directional cue). Taken together, our study shows that cavity orientation is an important feature of woodpecker cavities that is associated with variation in reproductive success and likely to be an adaptive trait of the RCW extended phenotype.

## Supplementary Information

Supplementary Information.

## Data Availability

Data are available in the Supplementary Information.
