# Supplementary material for: Effects of cavity orientation on nesting success inferred from long-term monitoring of the endangered red-cockaded woodpecker
Source: Sci Rep. 2022 Jul 8;12:11624. doi: 10.1038/s41598-022-15201-x (PMC9270470; doi:10.1038/s41598-022-15201-x)
Supplement: Supplementary file 1 — Supplementary Information. [file 41598_2022_15201_MOESM1_ESM.zip › data_RCW_SciRep_final/Legends_for_data_files.docx]

Data file 1: Data on population level cavity orientation, based on our own study and published records.

Data file S2: Data on individual cavity orientation from our study, including the stage of the cavity.

Data file S3: Data for our analysis of cavity orientation fitness effects.
